# Characterization of photovoltaics with In_2_S_3_ nanoflakes/*p*-Si heterojunction

**DOI:** 10.1186/1556-276X-9-32

**Published:** 2014-01-15

**Authors:** Yu-Jen Hsiao, Chung-Hsin Lu, Liang-Wen Ji, Teen-Hang Meen, Yan-Lung Chen, Hsiao-Ping Chi

**Affiliations:** 1 National Nano Device Laboratories, No. 27, Nanke 3rd Rd., Xinshi District, Tainan 74147, Taiwan; 2 Department of Chemical Engineering, National Taiwan University, Roosevelt Rd., Da-an District, Taipei 617, Taiwan; 3 Institute of Electro-Optical and Materials Science, National Formosa University, Wénhuà Rd., Huwei, Yunlin 632, Taiwan; 4 Department of Electronic Engineering, National Formosa University, Roosevelt Rd., Da-an District, Yunlin 632, Taiwan; 5 Department of Electrical Engineering, Nan Jeon Institute of Technology, No. 178, Chao-Zing Rd., Yanshui District, Tainan 737, Taiwan

**Keywords:** Heterojunction, Nanoflake, In_2_S_3_

## Abstract

We demonstrate that heterojunction photovoltaics based on hydrothermal-grown In_2_S_3_ on p-Si were fabricated and characterized in the paper. An n-type In_2_S_3_ nanoflake-based film with unique 'cross-linked network’ structure was grown on the prepared p-type silicon substrate. It was found that the bandgap energy of such In_2_S_3_ film is 2.5 eV by optical absorption spectra. This unique nanostructure significantly enhances the surface area of the In_2_S_3_ films, leading to obtain lower reflectance spectra as the thickness of In_2_S_3_ film was increased. Additionally, such a nanostructure resulted in a closer spacing between the cross-linked In_2_S_3_ nanostructures and formed more direct conduction paths for electron transportation. Thus, the short-circuit current density (*J*sc) was effectively improved by using a suitable thickness of In_2_S_3_. The power conversion efficiency (PCE, *η*) of the AZO/In_2_S_3_/textured p-Si heterojunction solar cell with 100-nm-thick In_2_S_3_ film was 2.39%.

## Background

Indium sulfide (In_2_S_3_) is one of the important semiconductor materials with direct bandgap and attracts intense interest due to its high photosensitivity, photoconductivity, and photocatalyst characteristics at ambient conditions [1-3]. In In_2_S_3_, there are three polymorphic forms: defect cubic structure α-In_2_S_3_, defect spinel structure β-In_2_S_3_, and higher-temperature-layered structure γ-In_2_S_3_[4]. Among them, β-In_2_S_3_ is an n-type semiconductor with superior photoelectric conversion function that can be employed in near-infrared to ultraviolet regions of solar energy absorption [5]. Hence, we may expect that β-In_2_S_3_ will act as a good absorber in heterojunction thin film solar cells [6]. On the other hand, In_2_S_3_ is a nontoxic semiconductor material which also offers potential advantage in process without Cd and Pb. A cell with ITO/PEDOT:PSS/In_2_S_3_:P3HT/Al structure has been fabricated by Jia et al. [7], which showed the short-circuit current density (*J*sc) of 0.68 mA cm^-2^ and a power conversion efficiency of 0.04%.

In recent years, In_2_S_3_ thin films have been grown by a variety of deposition techniques such as chemical bath deposition (CBD) [8], thermal evaporation [9], solvothermal synthesis [10], and atomic layer chemical vapor deposition (ALCVD) [11]. Among them, chemical bath deposition is a desirable method because of its low cost, arbitrary substrate shapes, simplicity, and can be easily prepared in large areas. There have been many reports for the heterojunction solar cell with CBD grown In_2_S_3_. For example, In_2_S_3_ was used for the n-type buffer layer of CIGS solar cells [12]. Crystalline silicon solar cells are presently the predominant photovoltaic devices among various solar cells due to their higher photovoltaic conversion efficiency, and long-term stability [13]. Recently, Abd-El-Rahman and Darwish et al. reported a p-Sb_2_S_3_/n-Si heterojunction photovoltaic that was fabricated by using thermal evaporation technique [14], which showed *J*sc = 14.53 mA cm^-2^, fill factor = 0.32, and *η* = 4.65%.

In this study, the In_2_S_3_ thin films were deposited on a p-type silicon substrates via chemical bath deposition route. To our knowledge, works on In_2_S_3_ film deposited on textured Si-based solar cell by CBD are few. In addition, the advantages of chemical bath deposition process are low temperature and low-cost synthesis. This fact motivates this work which discusses the structure and electrical property of the AZO/In_2_S_3_/textured p-Si heterojunction devices.

## Methods

The In_2_S_3_ nanoflakes were prepared according to the CBD procedure reported by Bai et al. [15]. Typically, aqueous solutions of 0.025 M InCl_3_, 0.048 M thioacetamide (CH_3_CSNH_2_) (TAA), and 0.04 M acetic acid were mixed in a glass beaker under magnetic stirring. The beaker was maintained at a reaction temperature of 80°C using water bath. In addition, the samples of silicon wafer were cleaned using a standard wet cleaning process. Subsequently, KOH was diluted to isotropically etch the silicon wafer to form a surface with a pyramid texture [16].

The preparation process of In_2_S_3_/p-Si heterojunction solar cell was separated into three parts: First, the samples with 1.5 × 1.5-cm^2^ square were cut from a (100)-oriented p-type silicon wafer with *ρ* = 10 Ω cm and 200-μm thickness. For ohmic contact electrodes, we used the DC sputtering technique to deposit 2-μm-thick Al onto the back of the Si substrates, followed by furnace annealing at 450°C for 1 h in Ar ambient conditions to serve Al as the p-ohmic contact electrodes. Second, 50 ~ 400-nm-thick n-type In_2_S_3_ thin films were deposited on the prepared p-type Si substrates by chemical bath deposition route in order to form an In_2_S_3_/p-Si heterojunction structure. Finally, an AZO film and Al metal grid with thicknesses of 0.4 and 2 μm, respectively, were deposited by sputtering. The purpose of AZO deposition is to produce a transparent conductive film by RF magnetron sputtering using ZnO:Al (2 wt.% Al_2_O_3_) target with a purity of 99.99% with 300-W power. All devices with the same AZO thickness (approximately 400 nm) were deposited at the same conditions. The single-cell size of photovoltaic device is about 0.4 cm^2^.

The phase identification of materials was performed by X-ray powder diffraction (Rigaku Dmax-33, Tokyo, Japan). The morphology and microstructure were examined by high-resolution transmission electron microscopy (HR-TEM; Hitachi HF-2000, Tokyo, Japan). The absorption and reflectance spectra were measured at room temperature using a Hitachi U-4100 UV–Vis-NIR spectrophotometer. The current density-voltage measurements (Keithley 2410 SourceMeter, Cleveland, OH, USA) were obtained by using a solar simulator (Teltec, Mainhardt, Germany) with an AM 1.5 filter under an irradiation intensity of 100 mW cm^-2^.

## Results and discussion

**Figure 1:**
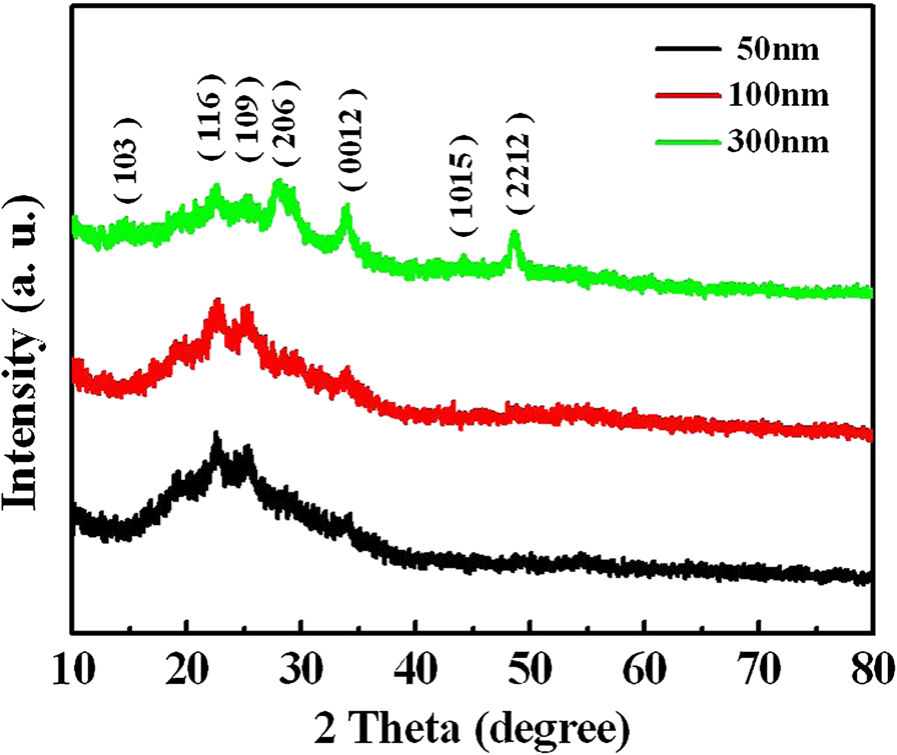
**XRD spectra of various thicknesses of In**_**2**_**S**_**3 **_**film synthesized using chemical bath deposition method at 80°C.**

XRD patterns of various In_2_S_3_ films with thicknesses of 50 to 300 nm are shown in Figure 1. The In_2_S_3_ films were formed directly from the amorphous precursors by using chemical bath deposition method. All of the peaks for various thicknesses were identified to be the tetragonal β-In_2_S_3_ phase (JCPDS card no. 25-0390) [17]. It can be seen that the crystallinity of In_2_S_3_ increases as the thickness of In_2_S_3_ film increases. The peaks of (206), (0012), and (2212) was observably seen while the thickness of In_2_S_3_ film was increased up to 300 nm. In this experiment, In^3+^ ions could form a variety of complexes in a solution. As InCl_3_ is dissolved in water, it is hydrolyzed and finally form In(OH)_3_. The possible chemical reactions for the synthesis of In_2_S_3_ nanocrystals can be expressed as following [18]:

(1) $$\begin{array}{l} {\left[\mathrm{In}{\left(H_{2}O\right)}_{6}\right]}^{3+}↔{{\left[\mathrm{In}{\left(H_{2}O\right)}_{5}\left(\mathrm{OH}\right)\right]}^{2}}^{+}\\ \;+H^{+}↔\mathrm{In}{\left(\mathrm{OH}\right)}_{3}+3H^{+} \end{array}$$

(2) $$\begin{array}{l} {\mathrm{CH}}_{3}{\mathrm{CSNH}}_{2}+H^{+}+2H_{2}O↔H_{2}S+{\mathrm{CH}}_{3}\mathrm{COOH}\\ \;+{{\mathrm{NH}}_{4}}^{+} \end{array}$$

(3) $$H_{2}S↔{\mathrm{HS}}^{-}+H^{+},{\mathrm{HS}}^{-}↔S^{2-}+H^{+}$$

(4) $$2{{\mathrm{In}}^{3}}^{+}+3S^{2-}\to {\mathrm{In}}_{2}S_{3}.$$

During the reaction processes, sulfide ions were slowly released from CH_3_CSNH_2_ and reacted with indium ions. Consequently, the In_2_S_3_ nanoflakes were formed via an *in situ* chemical reaction manner. Equation (4) indicates that In_2_S_3_ is produced by the reaction of S^2-^ and In^3+^.

**Figure 2:**
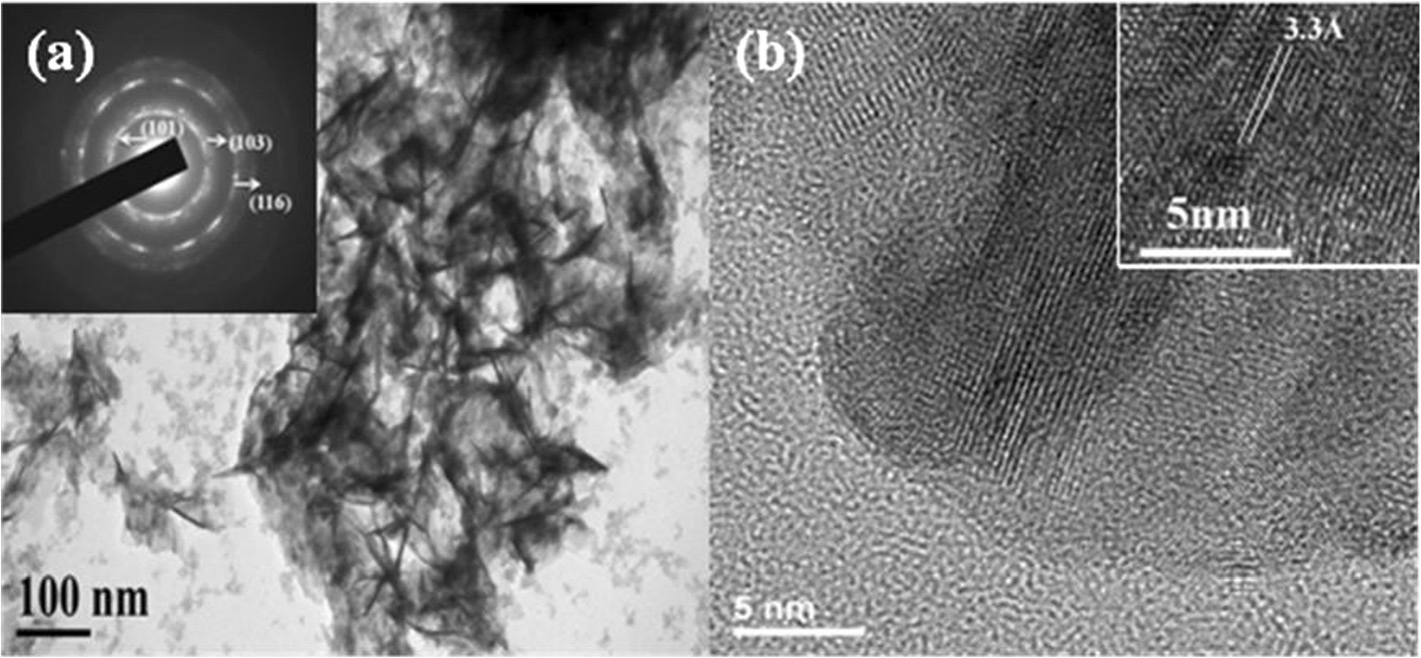
**TEM and HRTEM images of the In**_**2**_**S**_**3 **_**nanoflakes. (a)** TEM image of as-synthesized In_2_S_3_ nanoflakes and the electron diffraction pattern, **(b)** high-resolution TEM image of the nanocrystal.

TEM analysis provides further insight into the structural properties of as-synthesized nanoflakes In_2_S_3_. Figure 2a shows the low-magnification TEM image, and the nanoflakes can be clearly observed. The crystalline In_2_S_3_ nanoflakes are identified by electron diffraction (ED) pattern in the inset of Figure 2a, which exhibits diffusing rings, indicating that the In_2_S_3_ hollow spheres are constructed of polycrystalline In_2_S_3_ nanoflakes. The concentric rings can be assigned to diffractions from (101), (103), and (116) planes of tetragonal In_2_S_3_, which coincides with the XRD pattern. It is possible that the assembled effect arising from the nanocrystals results in the decrease of surface energy. A representative HRTEM image for such a tetragonal In_2_S_3_ nanostructure is shown in Figure 2b. It was found the interplanar distance of the crystal fringe is 3.3 Å, corresponding to the spacing of the (109) plane of tetragonal In_2_S_3_[19].

**Figure 3:**
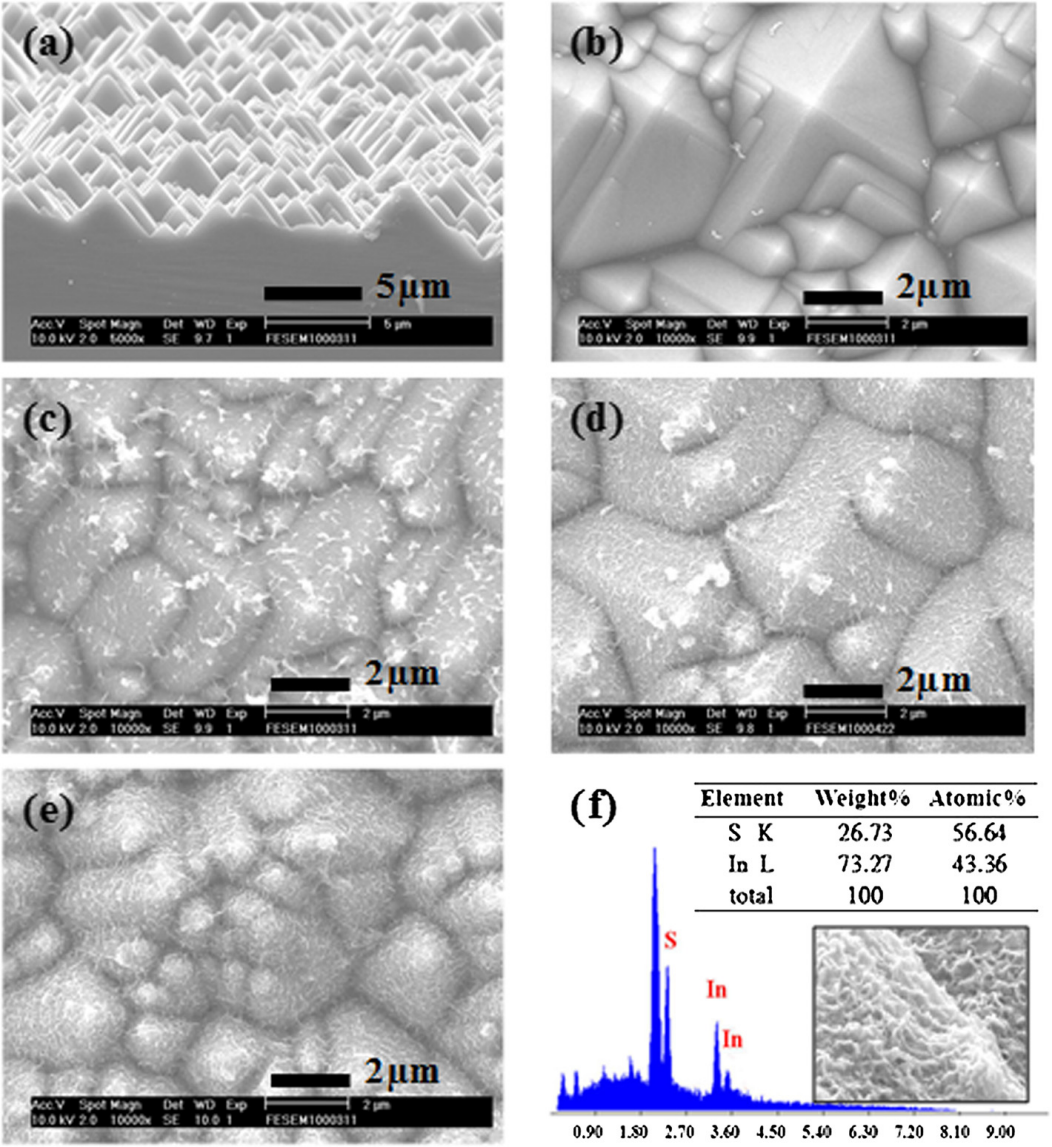
**SEM images of thep-Sisubstrate and an EDX analysis of the In**_**2**_**S**_**3 **_**film.(a)** Side-view and **(b)** top-view SEM images of the textured p-Si substrate, and **(c)** 50-nm, **(d)** 100-nm, and **(e)** 300-nm thick In_2_S_3_ films onto the textured p-Si. **(f)** EDX analysis of the In_2_S_3_ film, and the inset is a high-magnitude SEM image.

Figure 3a,b shows the side-view and top-view SEM images of the textured p-Si substrate by using wet etching process. The uniform pyramids had been made on the surface of the p-Si, which was defined as the anti-reflective structures for incident sunlight. The various thicknesses of In_2_S_3_ films were grown on the surface of the textured p-Si substrate; the thicknesses of the In_2_S_3_ films were about 50, 100, and 300 nm, respectively, as shown in Figure 3c,d,e. The images of the In_2_S_3_/textured p-Si substrate exhibit a rough surface. The EDS line profiles indicate that the film consists of indium and sulfur. The atomic concentrations of In = 56.6% and S = 43.4% are calculated from the EDS spectrum, as shown in Figure 3f. The In_2_S_3_ films were grown not only in the lateral direction, but also randomly in the vertical direction. In the inset of Figure 3f, we can see that the surface of the In_2_S_3_ film is with a cross-linked network structure.

**Figure 4:**
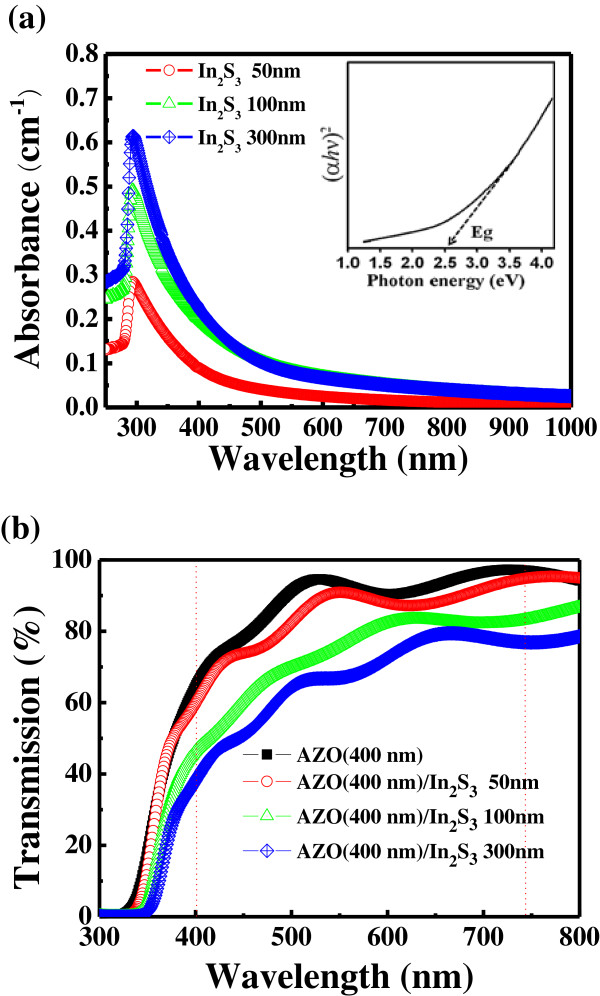
**Absorption and transmission spectra. (a)** Absorption spectra of the various thicknesses of the In_2_S_3_ film measured at room temperature. The inset shows a function of photon energy. **(b)** The transmission spectra of 400-nm-thick AZO deposited on the In_2_S_3_ film with various thicknesses.

We have measured the UV–Vis absorption spectra of the various thicknesses of the In_2_S_3_ film and estimated the bandgap energy from the absorption onset of data curves in Figure 4a. For a direct bandgap semiconductor, the absorbance in the vicinity of the onset due to the electron transition is given by

(5) $$\alpha =\frac{C{\left(\mathrm{h\nu}-E_{g}\right)}^{1/2}}{\mathrm{h\nu}},$$

where *α* is the absorption coefficient, *C* is the constant, *hν* is the photon energy, and *E*_g_ is the bandgap energy. The inset of Figure 4a reveals the relationship of (*αhν*)^2^ and *hν* gives a bandgap energy of 2.5 eV by the extrapolation of the linear region. The result was similar to previous report that 120- and 68-nm thicknesses of thermal-evaporated tetragonal In_2_S_3_ are with the bandgap of 2.54 and 2.52 eV, respectively [20].

Figure 4b shows the transmittance spectra of the 400-nm-thick AZO films on In_2_S_3_ films with various thicknesses. While the pure 400-nm AZO film on the glass showed 90.2% of transmittance, the transmittance values of 400-nm-thick AZO on In_2_S_3_ with 50-, 100-, and 300-nm thickness were about 86.2%, 75.5%, and 68.6%, respectively. It can be seen that the transmittance is decreased as the thickness of In_2_S_3_ film increases.

**Figure 5:**
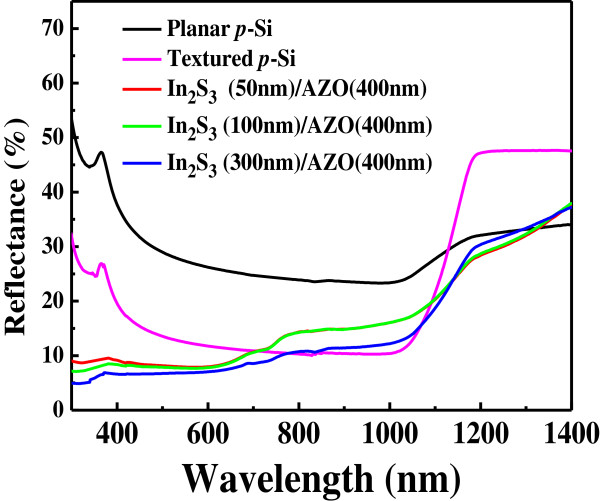
**Reflectance spectra of the planar p-Si, textured p-Si, and the In**_**2**_**S**_**3 **_**film with various thicknesses on textured p-Si substrate.**

Figure 5 shows the reflectance spectra of the planar p-Si, textured p-Si, and the In_2_S_3_ film with various thicknesses on textured p-Si substrate in the range of 200 ~ 1,100 nm. The average reflectance was about 11.3%, 10.9%, and 8.7% for the In_2_S_3_ film on the textured p-Si substrate with 50-, 100-, and 300-nm thicknesses, respectively. These values are lower than the average reflectance of planar p-Si and textured p-Si (32.0% and 16.2%, respectively). Therefore, the reflectance is obviously reduced by the nanoflake In_2_S_3_ and decreased as the thickness of In_2_S_3_ film increases. It could be attributed to the decreasing reflectance for In_2_S_3_ film at short wavelengths because the nanotexturization was on the surface [21].

**Figure 6:**
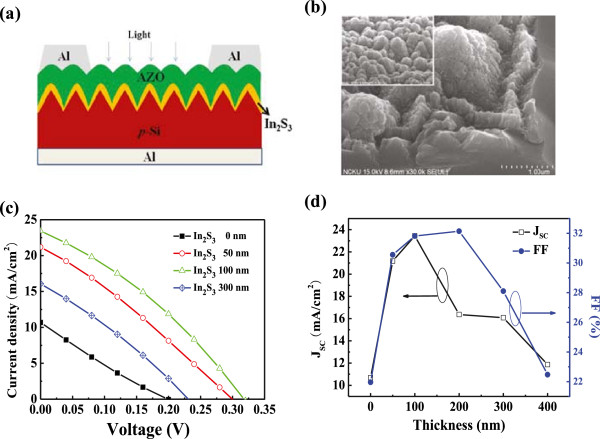
**Structure, SEM image,*J*-*V *characteristics, and *J*sc and FF of the heterojunction solar cells. (a)** Schematic structure of In_2_S_3_/textured p-Si heterojunction solar cell, **(b)** SEM image of AZO/In_2_S_3_/textured p-Si, **(c)***J*-*V* characteristics, and **(d)** the *J*sc and fill factor (F.F.) of the In_2_S_3_/p-Si heterojunction solar cell with various thicknesses of In_2_S_3_.

Figure 6a displays the schematic structure of the heterojunction solar cell in which the nanotextured In_2_S_3_/p-Si was the photoactive layer of such a device. Photovoltaic performance of the AZO/In_2_S_3_/p-Si heterojunction solar cell with various In_2_S_3_ thicknesses is given in Table 1. All samples for the electrical measurement were performed with AZO film of about 400 nm. Characterization of the AZO/In_2_S_3_ film deposited on the textured p-Si substrate was studied for the first time. Figure 6b shows a SEM image of an inclined angle of the AZO/In_2_S_3_/p-Si heterojunction structure. The AZO deposited on the In_2_S_3_ (100 nm)/p-Si substrate exhibits a well coverage and turns into a cylinder-like structure with a hemispherical top as shown in the inset of Figure 6b. The deposition thickness of the AZO was estimated to be 400 nm. Jiang et al. [22] revealed that they had fabricated the SnS/α-Si heterojunction photovoltaic devices, which the junction exhibited a typical rectified diode behavior, and the short-circuit current density was 1.55 mA/cm^2^. Hence, the AZO/In_2_S_3_/p-Si structure in the study was suitable for solar cell application.

**Table 1:** **Photovoltaic performance of the AZO/In**_**2**_**S**_**3**_**/p-Si heterojunction solar cell with various thicknesses of In**_**2**_**S**_**3**_

**Device**	***V***_**oc**_	***J***_**sc**_**(mA/cm**^**2**^**)**	**F.F. (%)**	**Efficiency (%)**
Non-In_2_S_3_	0.20	10.68	21.95	0.47
In_2_S_3_ (50 nm)	0.28	21.18	30.55	1.81
In_2_S_3_ (100 nm)	0.32	23.43	31.82	2.39
In_2_S_3_ (200 nm)	0.24	16.37	32.14	1.26
In_2_S_3_ (300 nm)	0.24	16.08	28.10	1.08

The photovoltaic condition is AM 1.5 G at 100-mW/cm^2^ illumination.

The current–voltage (*J*-*V*) characteristics of the fabricated photovoltaic devices were measured under an illumination intensity of 100 mW/cm^2^, as shown in Figure 6c. Such result shows that the short-circuit currents (*J*sc) were increased while the In_2_S_3_ films were deposited onto the p-Si. The power conversion efficiency (PCE) of the devices can be obviously improved from 0.47% to 2.39% by employing a 100-nm-thick In_2_S_3_ film. It was also found that the highest open-circuit voltage (*V*oc) and short-circuit current density are 0.32 V and 23.4 mA/cm^2^, respectively. Therefore, the optimum thickness of the In_2_S_3_ film is 100 nm, with PCE of 2.39%. When the thickness of the In_2_S_3_ film increases, the efficiency decreased because of the decrease in *J*sc and FF, as shown in Figure 6d. A similar phenomenon was also observed in the In_2_S_3_/CIGS heterojunction thin film solar cell [23]. It is possible that some defects on the interface of the AZO/In_2_S_3_/p-Si heterojunction with thicker In_2_S_3_ films will decrease the PCE. The cell performance improved markedly as the thickness of the In_2_S_3_ layer was increased to 100 nm. This improved cell performance is attributed to the reduction of possible shunt paths by the inclusion of a high-resistivity In_2_S_3_ buffer layer between the transparent conducting ZnO:Al and the p-Si layers. The cell performance, however, deteriorated in devices with 200- and 300-nm-thick In_2_S_3_ layers since the series resistance of the solar cell increased due to the high resistance of the In_2_S_3_ layer. Therefore, the 100-nm In_2_S_3_ sample shows the best performance.

## Conclusions

In summary, we have successfully synthesized the nanoflake In_2_S_3_ by a chemical bath deposition route in the study. The well-crystallized single phase of tetragonal In_2_S_3_ that can be obtained at 80°C and deposited on *p*-Si substrate was investigated for the first time. The visible light absorption edge of the as-grown In_2_S_3_ film corresponded to the bandgap energy of 2.5 eV by UV–Vis absorption spectra. It can be seen that the lower reflectance spectra occurred while the thickness of In_2_S_3_ film on the textured p-Si was increased. The photovoltaic characteristics of the AZO/In_2_S_3_/textured p-Si heterojunction solar cells with various In_2_S_3_ thicknesses were also given in the investigation, and the PCE of such device with 100-nm-thick In_2_S_3_ film is 2.39% under 100-mW/cm^2^ illumination.

## Abbreviations

FE-SEM: Field emission scanning electron microscopy; HRTEM: High-resolution transmission electron microscopy; UV–Vis: Ultraviolet–visible absorption spectra; PCE: Power conversion efficiency.

## Competing interests

The authors declare that they have no competing interests.

## Authors’ contributions

YJH and LWJ carried out the design of the study and drafted this manuscript. CHL and THM conceived of the study and participated in its design and coordination. YLC and HPC carried out the preparation of the samples and characteristic measurements. All authors read and approved the final manuscript.

## Authors’ information

YJH was born in Tainan, Taiwan, in 1976. He received his Ph.D. degree in Materials Science and Engineering from the National Cheng Kung University, Tainan, Taiwan, in 2007. He is an Associate Researcher in the National Nano Device Laboratories, Tainan. His current research interests include organic solar cell, thin film solar cell, and functional nanocrystals synthesis. CHL was born in Taipei, Taiwan. He earned his B.S. degree from the Department of Chemical Engineering, National Taiwan University, Taipei, Taiwan, in 1983, and his M.S. and Ph.D. degrees in Inorganic Materials from the Institute of Electrical Engineering, Tokyo and the Institute of Technology, Tokyo, Japan, in 1988 and 1991, respectively. Currently, he is a Full Professor in the Department of Chemical Engineering, National Taiwan University, Taipei, Taiwan. His current research interests include nanosized electronic and electro-optical materials and thin film processing. He is a recipient of the Outstanding Research Award from the National Science Council, Taiwan in 2010. LWJ was born in Taipei, Taiwan, in 1965. He received his B.S. degree in Physics, his M.S. degree in Material Science, and his Ph.D. degree in Electrical Engineering from the National Cheng Kung University (NCKU), Tainan, Taiwan. Currently, he is a Full Professor in the Institute of Electro-Optical and Materials Science, National Formosa University (NFU), Yunlin, Taiwan. From August 2005 to July 2006, he served as the Director of the R&D Center for Flat Panel Display Technology, NFU. His current research interests include semiconductor physics, optoelectronics, and nanotechnology. He is currently the Editor-in-Chief of the Journal of Science and Innovation (ISSN 2078-5453), the Taiwanese Institute of Knowledge Innovation (TIKI). LWJ was a recipient of the Research Award from Lam Research Taiwan Co., Ltd., Taiwan, in 2004. He has won a Gold Award in Seoul International Invention Fair 2013 (SIIF2013, November 29 to December 2, 2013), Seoul, South Korea. THM was born in Tainan, Taiwan, in 1967. He received his B.S. degree from the Department of Electrical Engineering, National Cheng Kung University, Tainan, Taiwan, in 1989, and his M.S. and Ph.D. degrees from the Institute of Electrical Engineering, National Sun Yat-Sen University, Kaohsiung, Taiwan, in 1991 and 1994, respectively. Currently, he is a Professor in the Department of Electronic Engineering, National Formosa University, Yunlin, Taiwan. His current research interests include semiconductor physics, optoelectronic devices, and nanotechnology. YLC received his M.S. degrees from the Institute of Electro-Optical and Materials Science, National Formosa University, Yunlin, in 2011. His current research interests include optoelectronic devices and growth of semiconductor nanostructures. HPC was born in Tainan, Taiwan, in 1964. He earned his B.S. degree from the Department of Electrical Engineering, Feng Chia University, Taichung, Taiwan, in 1990, and his M.S. and Ph.D. degrees in Electrical Engineering from the National Cheng Kung University (NCKU), Tainan, Taiwan, in 1993 and 2005, respectively. Currently, he is an Associate Professor in the Department of Electrical Engineering, Nan Jeon Institute of Technology, Tainan, Taiwan.
